# The Hahnemann Club of Toronto

**Published:** 1888-01

**Authors:** 


THE HAHNEMANN CLUB, of Toronto, was recently organized by Drs. John Hall, Sr., J. D. Tyrrell, E. T. Adams, A. Beattie Eadie, W. J. Hunter Emory, and L. H. Evans. The preamble and resolutions adopted are essentially those of the I. H. A. and have the true ring. With such purposes as these resolutions express and with such membership, one cannot fail to expect much of this new Club, and we feel confident our expectations will not be disappointed. The formation of Hahnemannian Associations and Clubs is certainly an excellent indication of the awakening interest in true Homœopathy. Success to them all.

